# Role of nuclear receptors *NlHR3* and *NlFTZ-F1* in regulating molting and reproduction in *Nilaparvata lugens* (stål)

**DOI:** 10.3389/fphys.2023.1123583

**Published:** 2023-03-15

**Authors:** Kailong Li, Kanghong Liu, Xing Wang, Mingyong Ma, Xiangwen Luo, Wuying Chen, Ang Chen, Zhaopu Peng, Deyong Zhang

**Affiliations:** Hunan Plant Protection Institute, Hunan Academy of Agricultural Science, Changsha, China

**Keywords:** *HR3*, *FTZ-F1*, *Nilaparvata lugens*, molting, reproduction

## Abstract

The nuclear receptors *HR3* and *FTZ-F1* are highly conserved and function to regulate molting and reproduction in both hemimetabolous and holometabolous insects. However, their roles in *Nilaparvata lugens* are largely unknown. In the present study, we discover that *NlHR3* and *NlFTZ-F1* are activated in the nymph stages by ecdysone signaling. Transcription disruption of *NlHR3* and *NlFTZ-F1* expression prevents nymph ecdysis and metamorphosis, which leads to abnormal appearance, malformed ovaries, and lethal phenotypes. In addition, we demonstrate that *NlHR3* and *NlFTZ-F1* regulate molting and reproduction by interacting with the intrinsic 20E and JH signaling pathways. Our work offers a deep insight into the action mechanisms of *HR3* and *FTZ-F1* in insects. Moreover, *NlHR3* and *NlFTZ-F1* could properly be exploited as potential target genes for developing RNAi-based pesticides to control *N. lugens*.

## 1 Introduction

The steroid hormone 20-hydroxyecdysone (20E) and the sesquiterpenoid juvenile hormone (JH) play key roles in insect growth and development. In the nucleus, 20E can bind to the heterodimeric complex of the ecdysone receptor (EcR)/ultraspiracle, triggering the expression of 20E-induced cascade genes, such as Broad-Complex (*Br-C*), hormone receptor 3 (*HR3*), *HR4*, ecdysone-induced protein 75B (*E75B*), and *FTZ-F1* (Pierceall et al., 1999; Swevers et al., 2002; Siaussat et al., 2004; Cruz et al., 2008; Liu et al., 2014; Li et al., 2015; Zhang et al., 2022). These ecdysone cascade genes are mostly nuclear receptor genes, having well-conserved DNA-binding domains and ligand-binding domains, and play a critical role in a variety of signaling and metabolic pathways, including the 20E signaling pathway (King-Jones and Thummel, 2005).

Hormone receptor 3 (*HR3*) belongs to the nuclear receptor superfamily, with a typical domain structure, including variable N-terminal domains (A/B), DNA-binding domains (C), hinge regions (D), and ligand-binding domains (E/F) (Nakagawa and Henrich, 2009). *HR3*s have been characterized in several species of *Lepidoptera* (Eystathioy et al., 2001), *Diptera* (Carney et al., 1997), *Blattaria* (Cruz et al., 2007), and *Coleoptera* (Tan and Palli, 2008; Xu et al., 2010; Zhao et al., 2018). DmHR3 plays an important role in enhancing molting and metamorphosis in *Drosophila melanogaster* (Lam et al., 1997; Lam et al., 1999). RNA interference (RNAi) causes developmental deficiency and mortality, indicating *HR3* is a crucial gene for molting and metamorphosis in several insects (Xu et al., 2010; Guo et al., 2015; Zhao et al., 2018).

*FTZ-F1* (*fushi tarazu* factor 1), a nuclear receptor-type transcription factor, was first identified during early embryogenesis as a transcription factor binding to the promoter of the pair-rule segmentation gene known as *fushi tarazu* (Lala et al., 1992). *FTZ-F1* was involved in molting and metamorphosis in *Drosophila* (Dubrovsky et al., 2011; Sultan et al., 2014), *Aedes aegypti* (Li et al., 2000), *Blattella germanica* (Cruz et al., 2008), *Spodoptera litura* (Tang et al., 2011), *Tribolium castaneum* (Tan and Palli, 2008), and *Leptinotarsa decemlineata* (Liu et al., 2014). The knockdown of *FTZ-F1* led to developmental arrest and phenotypic defects in these insects. The transcription of ecdysone biosynthetic genes, phantom and disembodied, could also respond to *FTZ-F1* to regulate ecdysone biosynthesis (Parvy et al., 2005). The knockdown of *LdFTZ-F1* inhibited the expression of ecdysone biosynthetic genes and reduced the titer of 20E, which eventually leads to pupation failure in *L. decemlineata* (Liu et al., 2014).

The 20E pulse triggers a transcriptional cascade composed of 20E early-response genes, such as hormone receptor 3 (*HR3*) and *HR4* (King-Jones and Thummel, 2005; Ruaud et al., 2010), and then induces a nuclear receptor factor, *FTZ-F1*, to play a developmental switch role (Dubrovsky, 2005; Zhang et al., 2021). In *D. melanogaster*, DmHR3 induced the expression of *βFTZ-F1* by directly binding to DmHR3-binding sites and transmitted a 20E signal cascade (Parvy et al., 2014). All these results indicated that *HR3* and *FTZ-F1* play a crucial role in insect growth and development, and there are interactions between them and the 20E signaling pathway.

The brown planthopper, *Nilaparvata lugens* (Stål), is one of the most destructive pests that feed on rice plants, causing a significant threat to rice production of China and other Asian countries (Cheng et al., 2003; Heong and Hardy, 2009). Although the molecular functions of *HR3* and *FTZ-F1* at molting and metamorphosis stages in holometabolous insects have been well investigated, their role in *N. lugens* and the relationship between *NlHR3*, *NlFTZ-F1*, and the 20E signaling pathway remain unclear. In the present study, we cloned *HR3* and *FTZ-F1* from *N. lugens* and analyzed their developmental and tissue-specific expression profiles. The relationship between *NlHR3*, *NlFTZ-F1*, and the 20E signaling pathway was also investigated through RNAi, the gene expression level, and measurements of survival rates. The results will aid the elucidation of insect molting and metamorphosis mediated by nuclear receptor genes and provide potential target genes for the development of RNAi insecticides to control the brown planthopper.

## 2 Materials and methods

### 2.1 Insect rearing

Brown planthoppers were reared on a susceptible rice (*Oryza sativa*) variety, Taichung Native 1 (TN1), cultivated at 27°C ± 0.5°C and 75% ± 5% relative humidity under a 14L: 10D (h) photoperiod, according to a previously described method (Li et al., 2018).

### 2.2 RNA extraction and cDNA synthesis

Total RNA was extracted with the TRIzol Total RNA Isolation Kit (Invitrogen, Carlsbad, CA, United States). The concentration and purity were measured with the NanoDrop 1000 spectrophotometer (Thermo Fisher Scientific, Rockford, IL, United States) and determined by agarose gel electrophoresis. cDNA was synthesized by using the ReverTra Ace qPCR RT Kit (Toyobo Co., Ltd., Osaka, Japan), following the manufacturer’s manual.

### 2.3 Molecular cloning and sequence analysis

Based on the published *N. lugens* genomic and transcriptomic data (Xue et al., 2014; Wan et al., 2015), *NlHR3 and NlFTZ-F1* homologies were identified and their sequences were confirmed by the reverse transcription polymerase chain reaction (RT-PCR) using primers, as shown in Supplementary Table S1. The PCR product was gel purified, ligated into the vector TOPO2.1 (Invitrogen, Carlsbad, CA), and transformed into *Escherichia coli* DH5α competent cells (Novagen, Darmstadt, Germany). A total of 10 recombinant plasmids from several independent subclones were fully sequenced on the Applied Biosystems 3730 automated sequencer (Foster City, CA) from both directions. The newly described transcript variants of *NlHR3* and *NlFTZ-F1* were submitted to GenBank.

ClustalW2 was used to perform a homologous sequence alignment of *HR3* and *FTZ-F1* proteins from *Nilaparvata lugens*, *Drosophila melanogaster*, *Tribolium castaneum*, *Bombyx mori*, *Apis mellifera*, *Aedes aegypti*, *Pediculus humanus corporis*, *Blattella germanica*, and *Acyrthosiphon pisum* (Larkin et al., 2007). The conserved domains were predicted by using the Simple Modular Architectural Research Tool (SMART; http://smart.embl-heidelberg.de/) and InterPro: protein sequence analysis and classification (http://www.ebi.ac.uk/interpro/).

### 2.4 Developmental- and tissue-specific expression profiles of *NlHR3* and *NlFTZ-*F1

For temporal expression analysis of *NlHR3* and *NlFTZ-F1*, total RNA samples were prepared from eggs; first (N1), second (N2), and third (N3) instar nymphs; each day of the fourth and fifth instar nymphs (N4D1, N4D2, N4D3, N5D1, N5D2, N5D3, and N5D4); and newly emerged (New-A) and 2-day-old female adults (AD2). To analyze the tissue-specific expression patterns, the different tissues were obtained by dissecting the fourth and fifth instar nymphs. The first group comprised the head (He), thorax (Th), and abdomen (Ab). The second group comprised the integument (In), wingbud (Wi), midgut (Mg), leg (Lg), and fat body (Fb). Quantitative real-time PCR (qRT-PCR) was conducted to estimate expression levels of *NlHR3* and *NlFTZ-F1* of various samples using internal control genes (*RSP15* and *RSP11*), according to the published methods (Wang et al., 2018). All the qRT-PCR primers are shown in Supplementary Table S1. There were three independent replications of each biological sample with three technical replicates. Data were analyzed by the 2^−ΔΔCT^ method (Livak and Schmittgen, 2001) and in accordance with the MIQE guidelines (Bustin et al., 2009).

### 2.5 20E treatment

The response of *NlHR3* and *NlFTZ-F1* to 20E application was determined by referring to previous methods (Gao et al., 2022). 20E was purchased from Sigma (Sigma-Aldrich, Shanghai, China). The fourth instar nymphs (0–12 h) were collected and treated with 0.2 μL 20E (0.1 μg/μL in acetone). After treatment with 20E, samples were taken at 6, 12, and 24 h after application to extract total RNA, and the expression of *NlHR3* and *NlFTZ-F1* was examined. Three replicates (10*3 nymphs, a total of 30 nymphs) were used to extract total RNA, and acetone was used as the control.

### 2.6 dsRNA synthesis and RNAi bioassay

The dsRNA synthesis and RNAi bioassay method were applied, as previously described (Li et al., 2018). The dsDNA fragments were amplified by RT-PCR using specific primers (Supplementary Table S1) and used as templates to synthesize dsRNA using the MEGAscript T7 High-Yield Transcription Kit (Ambion, Austin, TX, United States). The quality and concentration of the dsRNA were detected and kept at −80°C until further use. The dsRNA targeting the gene-encoding green fluorescence protein (dsGFP) served as a negative control.

RNA interference (RNAi) was performed by the FemtoJet microinjector (Eppendorf), as previously reported (Wang et al., 2018). Approximately 200 ng and 400 ng dsRNA were microinjected into each individual of the third and fifth instar nymphs, respectively (Xu et al., 2015). A total of 250 nymphs (10 replicates) were used for each treatment, including three survival evaluation replicates, three phenotypic evaluation replicates, three qRT-PCR verification replicates, and one backup replicate. QRT-PCR verification was conducted 3 days after injection.

### 2.7 Data analyses

Data were analyzed using Student’s *t*-test (the difference between two samples), Tukey’s test (the difference among three or more samples), and analysis of variance (ANOVA) by data processing system software (Tang and Zhang, 2013).

## 3 Results

### 3.1 Identification of the *HR3* and *FTZ-F1* transcripts of *N. lugens*

The cDNA of putative *HR3* and *FTZ-F1* transcripts in *N. lugens* was cloned and submitted to GenBank (OP937347, OP937348, KU928171.1, and KU928171.1). We identified two transcript variants of *HR3* (*NlHR3a* and *NlHR3b*) that encoded 520 and 462 amino acid residues and two transcript variants of *FTZ-F1* (*NlFTZ-F1a* and *NlFTZ-F1b*) that encoded 609 and 633 amino acid residues. The *HR3* and *FTZ-F1* family proteins contain two conserved nuclear receptor functional domains, among which the DNA-binding domain (DBD) at the amino terminal and the ligand-binding domain (LBD) at the carboxy terminal are highly conserved (Supplementary Figures S1A, S2A). The phylogenetic tree analysis showed that *NlHR3* has a close relationship with that of *A. pisum*, and *NlFTZ-F1* has a close relationship with that of *A. pisum* and *P. humanus* (Supplementary Figures S1C, S2C).

### 3.2 Expression profiles of *NlHR3* and *NlFTZ-F1*

**FIGURE 1:**
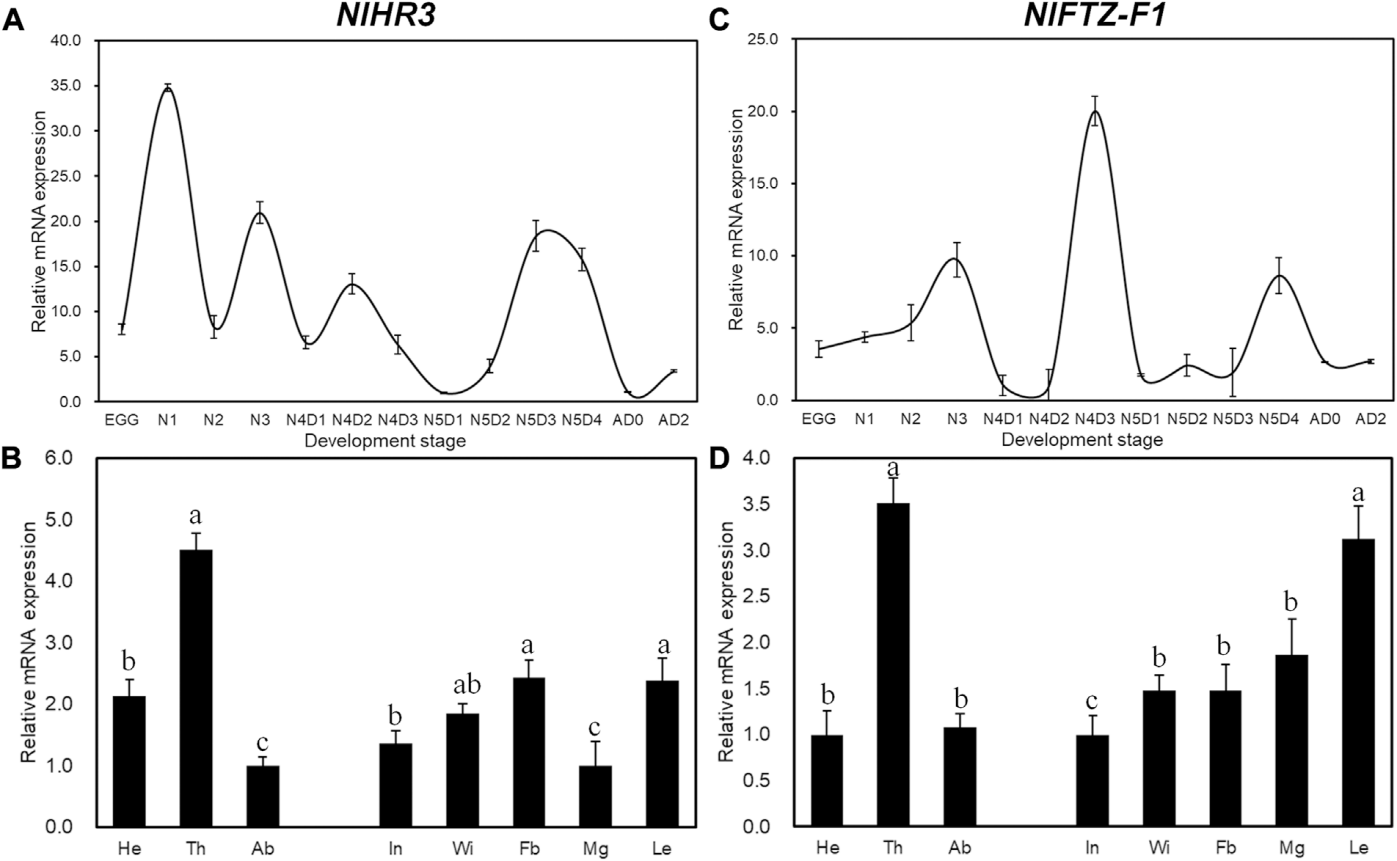
Temporal and spatial expression profiles of *NlHR3* and *NlFTZ-F1.* **(A, C)** Relative expression levels of *NlHR3* and *NlFTZ-F1* at different developmental stages. N1 (the first instar nymph) to N5D4 (day 4 of the fifth instar nymph), New-A (newly emerged female adults), and AD2 (2-day-old female adults). **(B, D)**: *NlHR3* and *NlFTZ-F1* relative expression levels of the head (He), thorax (Th), abdomen (Ab), integument (In), wingbud (Wi), midgut (Mg), leg (Le), and fat body (Fb) of nymphs (fourth–fifth instar). The standard error (SE) was determined from three independent biological replicates, each with three technical replications. Different letters indicate a significant difference at a *p*-value < 0.05 (Tukey’s test).

Developmental expression analysis showed that the transcript levels of *NlHR3* were the highest in the first instar period (Figure 1A), while *NlFTZ-F1* had two expression peaks at the later stages of the fourth and fifth instar periods (Figure1C). The expression profiles of *NlHR3* and *NlFTZ-F1* in different tissues are similar and relatively higher in the thorax and appendage of the nymphs (Figures 1B,D). The spatiotemporal data are compatible with the common idea that *NlHR3* and *NlFTZ-F1* play a vital role in the 20E signaling pathway.

### 3.3 Transcriptional responses to 20E

**FIGURE 2:**
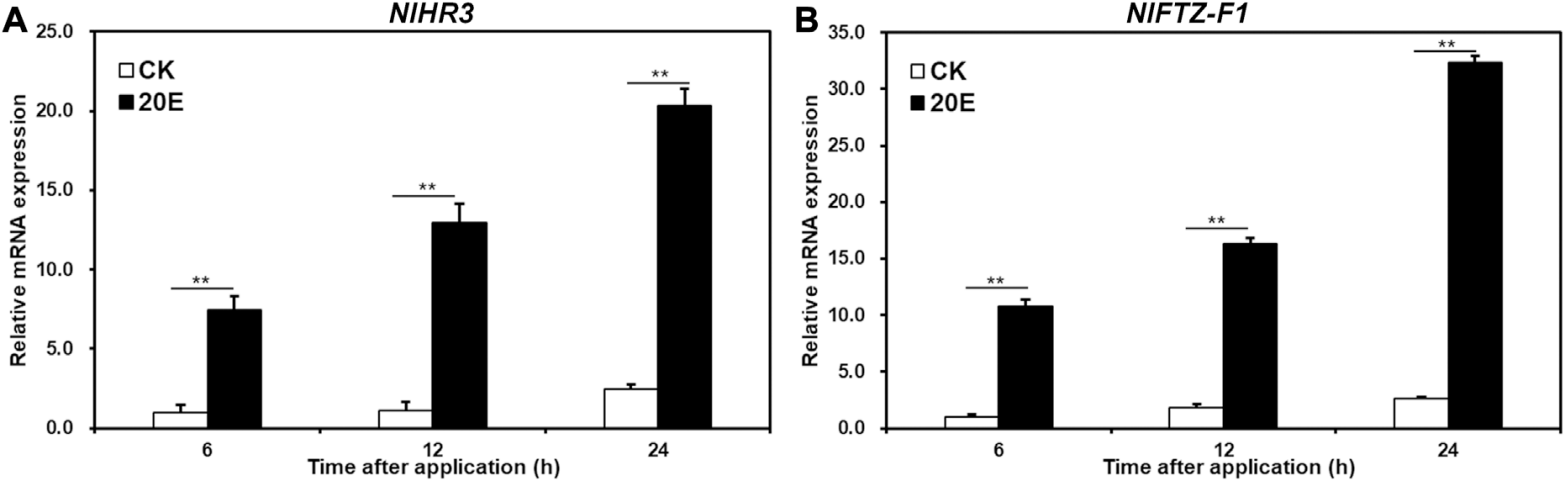
Transcriptional responses of *NlHR3* and *NlFTZ-F1* to the 20E signaling pathway. **(A)** Effect of 20E on the expression of *NlHR3*. **(B)** Effect of ecdysone on the expression of *NlHR3*. SE was determined from three independent biological replicates, each with three technical replications. ^**^ indicates a significant difference at a *p*-value < 0.01 (Student’s *t*-test).

In order to test whether *NlHR3* and *NlFTZ-F1* are induced by 20E, the fourth instar nymphs were treated with 20E or acetone (control) for 6, 12, and 24 h. The expression of *NlHR3* significantly increased by 7.4, 11.4, and 8.3 fold (*p*-value < 0.01) after 6, 12, and 24 h of 20E application, respectively, compared to the acetone control (Figure 2A), while the expression of *NlFTZ-F1* significantly increased 10.7-, 8.9-, and 12.1-fold (*p*-value < 0.01) (Figure 2B).

### 3.4 Regulation network between *HR3*, *FTZ-F1*, and the hormone signaling pathway

**FIGURE 3:**
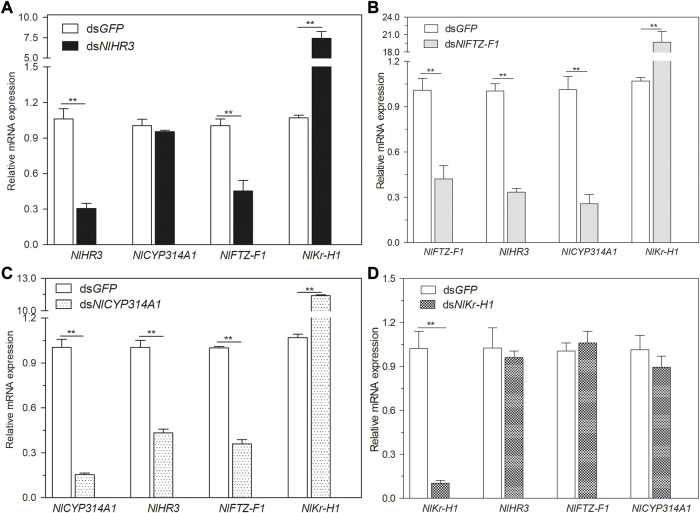
Relationship between *NlHR3*, *NlFTZ-F1*, and the hormone signaling pathway. **(A)** Downregulation of *NlHR3* affected 20E and JH signaling pathway gene expression. **(B)** Downregulation of *NlFTZ-F1* affected 20E and JH signaling pathway gene expression. **(C)** Downregulation of *NlCYP314A1* affected *NlHR3* and *NlFTZ-F1* expression. **(D)** Downregulation of *NlKr-H1* did not affect *NlHR3* and *NlFTZ-F1* expression. The SE was determined from three independent biological replicates, each with three technical replications. ^**^ indicates a significant difference at a *p*-value < 0.01 (Student’s *t*-test).

To explore the regulatory network between *HR3*, *FTZ-F1*, and the hormone signaling pathway, dsRNA of *NlHR3*, *NlFTZ-F1*, *NlCYP314A*, and *NlKr-h1* (Krüppel homolog 1) were injected into the third instar nymphs. Then, the transcript levels of *NlHR3*, *NlFTZ-F1*, *NlCYP314A*, and *NlKr-h1* were measured 3 days later. In the nymphs injected with ds*NlHR3*, the transcript levels of *NlCYP314A* were not significantly altered, but *NlFTZ-F1* was significantly decreased by 54.8% (*p*-value < 0.01) and *NlKr-h1* was significantly increased by 739.7% (*p*-value <0.01) compared to the dsGFP control (Figure 3A).

In the nymphs injected with ds*NlFTZ-F1*, the transcript levels of *NlCYP314A* and *NlHR3* were significantly decreased by 74.2% and 80.3% (*p*-value <0.01), respectively, and *NlKr-h1* was significantly increased by 1771.3% (*p*-value <0.01) (Figure 3B). In the nymphs injected with ds*NlCYP314A* (an ecdysone synthesis key gene), the transcript levels of *NlHR3* and *NlFTZ-F1* were significantly decreased by 56.6% and 64.1% (*p*-value <0.01), respectively (Figure 3C), while in the nymphs injected with ds*NlKr-h1* (a juvenile hormone response key gene), the transcript levels of *NlHR3* and *NlFTZ-F1* were not significantly different from that of the control (Figure 3D). Ecdysone receptor (EcR) is the starting point of an ecdysone cascade reaction and the upstream of *HR3* and *FTZ-F1*. We analyzed the expression level of *NlEcR*, but there was no significant difference after the knockdown of *NlHR3* and *NlFTZ-F1* (Supplementary Figure S3).

In summary, the transcript levels of 20E biosynthetic and cascade regulated genes were decreased significantly by silencing *NlFTZ-F1*, while only the ecdysone cascade regulated genes were decreased significantly by silencing *NlHR3*. The transcript level of the JH response key gene was increased significantly by silencing *NlHR3* and *NlFTZ-F1*. The transcript level of *NlFTZ-F1* was decreased significantly by silencing *NlHR3*.

### 3.5 Effect of expression silencing of *NlHR3* and *NlFTZ-F1* on nymph–nymph molting

**FIGURE 4:**
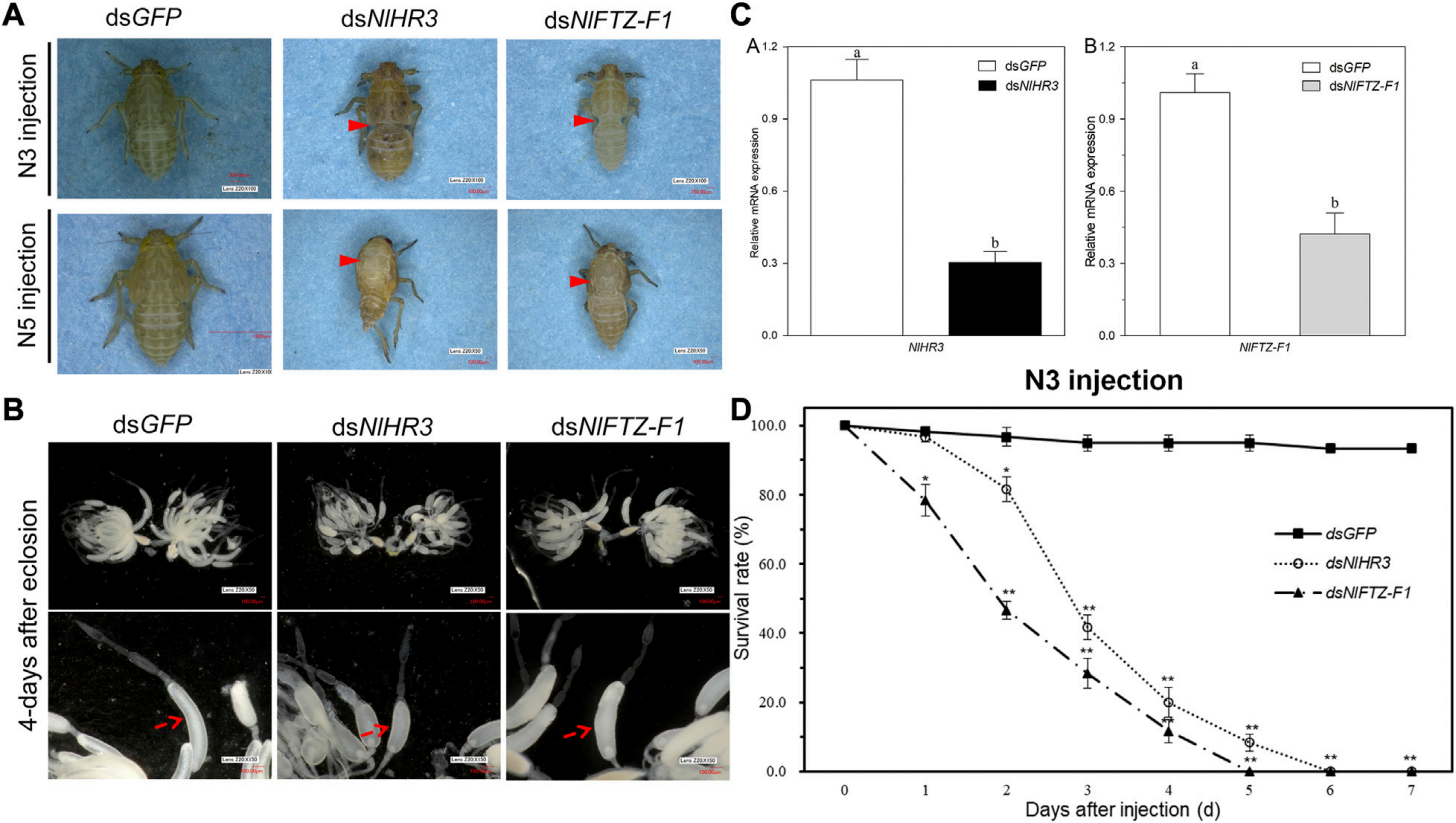
Phenotypes and the survival rate of *N. lugens* subjected to ds*NlHR3* and ds*NlFTZ-F1* injection. **(A)** Abnormal appearance of the phenotype of *N. lugens* subjected to ds*NlHR3* and ds*NlFTZ-F1* injection. **(B)** Abnormal appearance of the ovary phenotype of *N. lugens* subjected to ds*NlHR3* and ds*NlFTZ-F1* injection. **(C)** Relative expression level of *NlHR3* and *NlFTZ-F1* subjected to ds*NlHR3* and ds*NlFTZ-F1* injection in the third instar nymphs. **(D)** Survival rate of *N. lugens* subjected to ds*NlHR3* and ds*NlFTZ-F1* injection in the third instar nymphs. The SE was determined from three independent biological replicates, each with three technical replications. Different letters indicate a significant difference at a *p*-value < 0.05 (Student’s *t*-test). ^*^ and ^**^ indicate a significant difference at a *p*-value < 0.05 or < 0.01 (Student’s *t*-test).

In order to investigate the role of *NlHR3* and *NlFTZ-F1* in nymph–nymph molting, ds*NlHR3* and ds*NlFTZ-F1* from the common region (marked with black lines in Supplementary Figure S1) were synthesized *in vitro* and injected into the third instar nymphs. Compared to the control, the transcript levels of *NlHR3* and *NlFTZ-F1* were significantly decreased by 69.6% and 57.8% (*p*-value <0.01), respectively, 48 h after injecting with ds*NlHR3* (Figure 4D).

Upon injecting with dsRNA into the early third instar nymphs, the knockdown of *NlHR3* resulted in a significantly lower survival rate (81.7%; *p*-value < 0.05), which started on the second day after dsRNA injection (Figure 4D). The development of these nymphs was inhibited on the third instar stage, and all individuals (*n* = 60 out of 60) were unable to molt normally into the fourth instar stage and eventually died on the sixth day after dsRNA injection (Figures 4A,D). The knockdown of *NlFTZ-F1* resulted in a significantly lower survival rate (78.3%; *p*-value < 0.05) which started on the first day after dsRNA injection (Figure 4D). The insects injected with dsRNA, targeting *NlFTZ-F1*, exhibited lethal phenotypes at approximately 3 days after injection, and the development was inhibited on the third nymph–nymph molting transition; as a result, the insect bodies became slender and extended. The insects then failed to shed their old cuticle and died (Figure 4A). In contrast, the ds*GFP*-treated nymphs successfully completed all nymph–nymph molting transition, and the survival rate on the seventh day after injection was 93.3% (Figures 4A,D). Furthermore, *NlFTZ-F1* silencing caused a lower survival rate than *NlHR3* from the second day after injection. The survival rate decreased to 0% on the fifth day after downregulating *NlHR3*, while all individuals died on the sixth day for downregulating *NlFTZ-F1* (Figure 4D).

### 3.6 Effect of expression silencing of *NlHR3* and *NlFTZ-F1* on the nymph–adult transition

In order to investigate the role of *NlHR3* and *NlFTZ-F1* in nymph–adult molting, dsRNAs were injected into the fifth instar nymphs. The transcript levels of *NlHR3* and *NlFTZ-F1* were significantly decreased from the controls. The knockdown of *NlHR3* and *NlFTZ-F1* resulted in a significantly higher mortality rate (42.7% and 53.3%, *p*-value < 0.05) on the fourth day after dsRNA injection, and these individuals were unable to molt normally into adults; the old cuticle of the notum was split open, and the insects then failed to shed their old cuticle and died (Figure 4A). The individuals that molt normally into female adults were dissected to observe the development of ovaries. The ovaries of female individuals injected with ds*NlHR3* and ds*NlFTZ-F1* were deficient of oocytes and abnormally formed. The oocytes of individuals injected with ds*NlHR3* were smaller than those of the ds*GFP*, and the egg shell of oocytes in individuals injected with ds*NlFTZ-F1* had no luster and elasticity (Figure 4B), while the ovaries of those injected with ds*GFP* were full of oocytes (banana-shaped), and the egg shell of oocytes had luster and elasticity (Figure 4B). No offspring was produced by female individuals injected with ds*NlHR3* and ds*NlFTZ-F1*.

## 4 Discussion

In the present study, we cloned and characterized *HR3* and *FTZ-F1* genes in *N. lugens*, and their temporal and spatial expression profiles were analyzed. This is the first report of splice variants of *FTZ-F1* in *N. lugens*. The *HR3* and *FTZ-F1* genes have splice variants that are widely distributed in insects, including *D. melanogaster* (Dubrovsky et al., 2011), *A. aegypti* (Li et al., 2000), *Manduca sexta* (Weller et al., 2001), *T. castaneum* (Tan and Palli, 2008), *B. germanica* (Cruz et al., 2008), and *L. decemlineata* (Liu et al., 2014). Many nuclear receptor factors are induced and temporally expressed in response to 20E (Liu et al., 2014; Sultan et al., 2014; Li et al., 2015; Zhang et al., 2022). Among them, *FTZ-F1* was expressed after a pulse of 20E during the late nymph stage in almost all organs and is essential for molting and metamorphosis. In *N. lugens*, *NlFTZ-F1* was remarkably induced before or right after each molt in the fourth and fifth instar nymph stages and was induced by 20E. This temporally specific expression was in concert with the cyclical fluctuation of the 20E titer detected in a previous study (Liu et al., 2014; Zhao et al., 2019; Zhang et al., 2022).

Our results showed that the knockdown of *NlHR3* and *NlFTZ-F1* in nymphs severely impaired nymph molting and metamorphosis, and RNAi nymph resulted in lethal phenotypes and abnormally formed ovaries. Ecdysones regulate molting and reproduction of *N. lugens* (Zhou et al., 2020). In this study, the expression of *NlHR3* and *NlFTZ-F1* was significantly increased after 20E treatment (Figure 2), suggesting *HR3* and *FTZ-F1* were induced by 20E (Liu et al., 2014; Guo et al., 2015). *NlCYP314A1* is a key gene of the 20E biosynthetic pathway and played an important role in the nymphal development (Li et al., 2017). By knocking down *NlCYP314A1* in *N. lugens*, *NlHR3* and *NlFTZ-F1* mRNA levels were decreased (Figure 3C). These data demonstrated that *NlHR3* and *NlFTZ-F1* can participate in the growth and development regulated by 20E. Similar results have been reported in other insects. The expression pattern of *DmHR3* is related to the titer of 20E in *D. melanogaster*, and 20E promotes the expression of *DmHR3* (Carney et al., 1997). In the larvae of *B. germanica*, 20E directly regulates the expression of *BgHR3* and ecdysis was inhibited after the knockdown of *BgHR3* (Cruz et al., 2008). In *L. decemlineata*, *LdHR3* regulated the pupation process by responding to 20E (Guo et al., 2015). *D. melanogaster* larvae died due to severe ecdysis defects, following silencing *DmFTZ-F1* (Yamada et al., 2000). In *B. germanica*, the knockdown of *BgFTZ-F1* led to growth retardation and molting failure (Cruz et al., 2008). Recently, the knockdown of *HaFTZ-F1* severely damaged larval ecdysis in *Helicoverpa armigera* (Zhang et al., 2021), and the knockdown of *HvFTZ-F1* impaired molting, pupation, and reproduction in *Henosepilachna vigintioctopunctata* (Liu et al., 2022). The result from this study also suggested that *NlHR3* and *NlFTZ-F1* could be a potential target for the RNAi-mediated pest control against *N. lugens* because of the high mortality rate after RNAi. However, the unintended effects of ds*NlHR3* and ds*NlFTZ-F1* on target and non-target organisms should be investigated before the commercialization of RNAi-based pest control (Pan et al., 2020; He et al., 2022).

Furthermore, the knockdown of *NlFTZ-F1* resulted in varying degrees of downregulations of *NlCYP314A1* (20E-induced biosynthetic genes) and *NlHR3* (20E-induced cascade genes) (Figure 3B). In *D. melanogaster*, the expression levels of two steroidogenic enzymes, phantom (Phm) and disembodied (Dib), were reduced in *DmFTZ-F1* mutant ring gland cells (Parvy et al., 2005). *BgFTZ-F1* regulates the onset of production of ecdysteroids at mid-nymphal stages in *B. germanica* (Cruz et al., 2008). RNAi-mediated knockdown of *LdFTZ-F1* significantly repressed the transcription of *LdPHM*, *LdDIB*, and *LdSHD*, lowered the 20E titer in *L. decemlineata* (Liu et al., 2014)*.* In *H. armigera*, the knockdown of *HaFTZ-F1* lowered the intrinsic 20E titer, and reduced the expression of ecdysone receptors and 20E cascade regulated genes *HaBrC* and *HaE75A* (Zhang et al., 2021). These results indicated that the knockdown of *FTZ-F1* resulted in irreversible changes to 20E biosynthetic and downstream cascade regulated genes, thereby impairing molting and metamorphosis. However, how *FTZ-F1* regulates 20E biosynthetic and cascade regulated genes requires further study.

*Kr-h1* is one of the crucial effectors that mediate the interactions between JH and 20E signaling pathways (He and Zhang, 2022). The expression level of *NlKr-H1* was significantly increased after the knockdown of *NlHR3* and *NlFTZ-F1*, suggesting that *NlKr-H1* was negatively regulated by *NlHR3* and *NlFTZ-F1* (Figures 3A,B). The knockdown of *NlHR3* and *NlFTZ-F1* resulted in the reduction of 20E biosynthetic gene expression. These results led us to hypothesize that *NlHR3* and *NlFTZ-F1* could negatively regulate the JH signaling pathway by affecting 20E biosynthetic genes and the 20E titer. On the other hand, the mutual inhibition of the 20E- and JH-response pathways plays an important role in both embryonic and postembryonic phases (Uyehara et al., 2017; Liu et al., 2018; Truman, 2019; He and Zhang, 2022). Therefore, the interference of these 20E cascade regulated genes would lead to the weakening of the 20E cascade and the enhancement of the JH cascade, disrupting the normal synergy and accurate action time of the two hormone pathways and ultimately affecting the molting, metamorphosis, and reproduction processes of the brown planthopper.

## 5 Conclusion

Taken together, this is the first detailed report which shows that the nuclear transcription factors *NlHR3* and *NlFTZ-F1* are involved in the regulation of molting and reproduction in *N. lugens*. *NlHR3* and *NlFTZ-F1* regulate molting and reproduction by mediating the intrinsic 20E and JH signaling pathways. These results will deepen our understanding of the action mechanisms of *HR3* and *FTZ-F1* in insects. In addition, *NlHR3* and *NlFTZ-F1* could properly be exploited as potential target genes for developing RNAi-based pesticides to control *N. lugens*.

## Data Availability

The original contributions presented in the study are included in the article/Supplementary Materials; further inquiries can be directed to the corresponding authors.

## Supplementary material

SUPPLEMENTARY FIGURE S1 Splice variant structure comparison. Sequence alignment and phylogenetic tree of HR3. **(A)** Prediction of conservative domains and comparison of different transcript structures of *NlHR3*. **(B)** *HR3*s from *NlHR3A* and *NlHR3B* (*Nilaparvata lugens*), *AaHR3* (*Aedes aegypti*, AAF36970), *AgHR3* (*Anopheles gambiae*, EAA14853), *AmHR3* (*Apis mellifera*, XP_006570914), *ApHR3X1* (*Acyrthosiphon pisum*, XP_003240875), *ApHR3X2* (*Acyrthosiphon pisum*, XP_008189753), *ApHR3X3* (*Acyrthosiphon pisum*, XP_008189754), *ApHR3X4* (*Acyrthosiphon pisum*, XP_008189756), *BmHR3* (*Bombyx mori*, NP_001037012), *BmHR3X1* (*Bombyx mori*, XP_012551821), *NvHR3* (*Nasonia vitripennis*, XP_008202385), *TcHR3X1* (*Tribolium castaneum*, XP_974561), *TcHR3X2* (*Tribolium castaneum*, XP_008195824), and *TcHR3X3* (*Tribolium castaneum*, XP_008195825). Amino acids with 100, > 80, and > 60% conservations are shaded in black, dark gray, and light gray, respectively. Gaps have been introduced to permit alignments. **(C)** Phylogenetic tree of *HR3s* of *N*. *lugens* and other insects constructed by the neighbor-joining method based on the amino acid sequence. The test of phylogeny was carried out using a bootstrap analysis of 1000 replications; bootstrap values > 50% are shown on each node of the tree.

SUPPLEMENTARY FIGURE S2 Splice variant structure comparison. Sequence alignment and phylogenetic tree of FTZ-F1. **(A)** Prediction of conservative domains and comparison of different transcript structures of *NlFTZ-F1*. **(B)** *FTZ-F1s* from *NlFTZ-F1a* and *NlFTZ-F1b* (*Nilaparvata lugens*), *PhFTZ-F1* (*Pediculus humanus corporis*, XP_002430379), *TcFTZ-F1* (*Tribolium castaneum*, EFA01263), *TcFTZ-F1X1* (*Tribolium castaneum*, XP_008191373), *TcFTZ-F1X2* (*Tribolium castaneum*, XP_008191374), *TcFTZ-F1X3* (*Tribolium castaneum*, XP_008191375), *ApFTZ-F1X1* (*Acyrthosiphon pisum*, XP_008183294), *ApFTZ-F1X2* (*Acyrthosiphon pisum*, XP_008183295), *BgFTZ-F1* (*Blattella germanica*, CAQ57670), *AaFTZ-F1a* (*Aedes aegypti*, XP_001654601), *AaFTZ-F1b* (*Aedes aegypti*, CAO79104), *AgFTZ-F1a* (*Anopheles gambiae*, EAA11812), *AgFTZ-F1b* (*Anopheles gambiae*, EAL39882), *BmFTZ-F1* (*Bombyx mori*, NP_001037528), *DmFTZ-F1* (*Drosophila melanogaster*, AAA28542), *DmFTZ-F1b* (*Drosophila melanogaster*, AAA28915), *LdFTZ-F1a* (*Leptinotarsa decemlineata*, AJF93908), and *LdFTZ-F1b* (*Leptinotarsa decemlineata*, AJF93909). Amino acids with 100, > 80, and > 60% conservations are shaded in black, dark gray, and light gray, respectively. Gaps have been introduced to permit alignments. **(C)** Phylogenetic tree of *FTZ-F1s* of *N*. *lugens* and other insects constructed by the neighbor-joining method based on the amino acid sequence. The test of phylogeny was carried out using a bootstrap analysis of 1000 replications; bootstrap values > 50% are shown on each node of the tree.

SUPPLEMENTARY FIGURE S3 Effect of the knockdown of *NlHR3* and *NlFTZ-F1* on the expression of NlEcR. **(A)** Effect of knockdown of *NlHR3* on the expression of NlEcR. **(B)** Effect of knockdown of *NlFTZ-F1* on the expression of NlEcR. SE was determined from three independent biological replicates, each with three technical replications.

SUPPLEMENTARY TABLE S1 Primers used for RT-PCR, dsRNA synthesis, and qRT-PCR.
