# Supplementary material for: Role of nuclear receptors NlHR3 and NlFTZ-F1 in regulating molting and reproduction in Nilaparvata lugens (stål)
Source: Front Physiol. 2023 Mar 15;14:1123583. doi: 10.3389/fphys.2023.1123583 (PMC10050704; doi:10.3389/fphys.2023.1123583)
Supplement: Supplementary file 1 [file DataSheet1.DOCX]

**Supplementary Materials:**


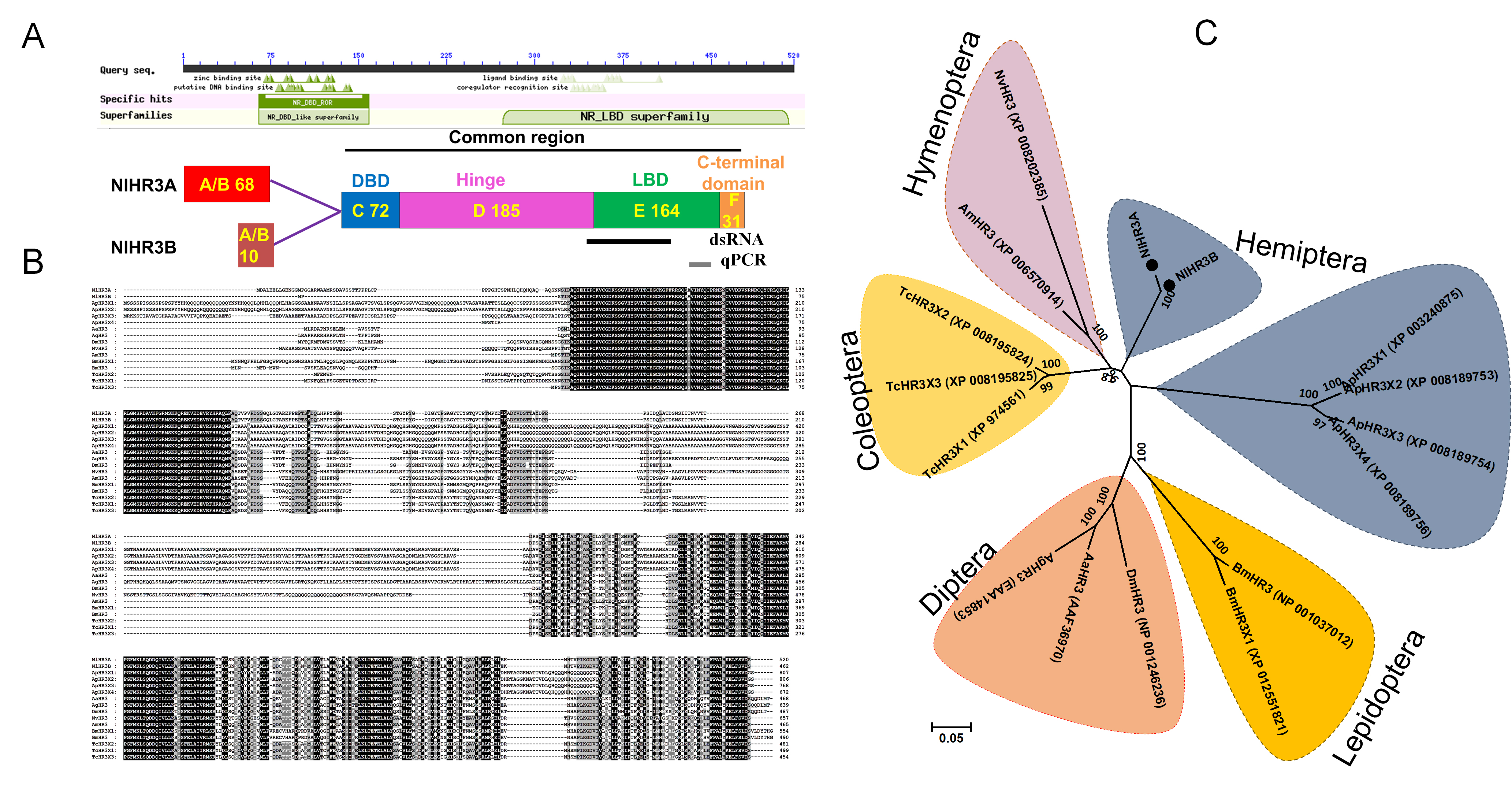


**Figure S1. Splice variants structure comparison, Sequence alignment and Phylogenetic tree of HR3.** (A) The prediction of conservative domains and comparison of different transcript structures of NlHR3. (B) HR3s from NlHR3A and NlHR3B (*Nilaparvata lugens*), AaHR3 (*Aedes aegypti*, AAF36970), AgHR3 (*Anopheles gambiae*, EAA14853), AmHR3 (*Apis mellifera*, XP_006570914), ApHR3X1 (*Acyrthosiphon pisum*, XP_003240875), ApHR3X2 (*Acyrthosiphon pisum*, XP_008189753), ApHR3X3 (*Acyrthosiphon pisum*, XP_008189754), ApHR3X4 (*Acyrthosiphon pisum*, XP_008189756), BmHR3 (*Bombyx mori*, NP_001037012), BmHR3X1 (*Bombyx mori*, XP_012551821), NvHR3 (*Nasonia vitripennis*, XP_008202385), TcHR3X1 (*Tribolium castaneum*, XP_974561), TcHR3X2 (*Tribolium castaneum*, XP_008195824), TcHR3X3 (*Tribolium castaneum*, XP_008195825). Amino acids with 100, >80, and >60% conservations are shaded in black, dark gray, and light gray, respectively. Gaps have been introduced to permit alignment. (C) Phylogenetic tree of HR3s of *N.lugens* and other insects constructed by neighbor-joining method based on amino acid sequence. The test of phylogeny was carried out using a bootstrap analysis of 1000 replications, bootstrap values > 50% are shown on each node of the tree.


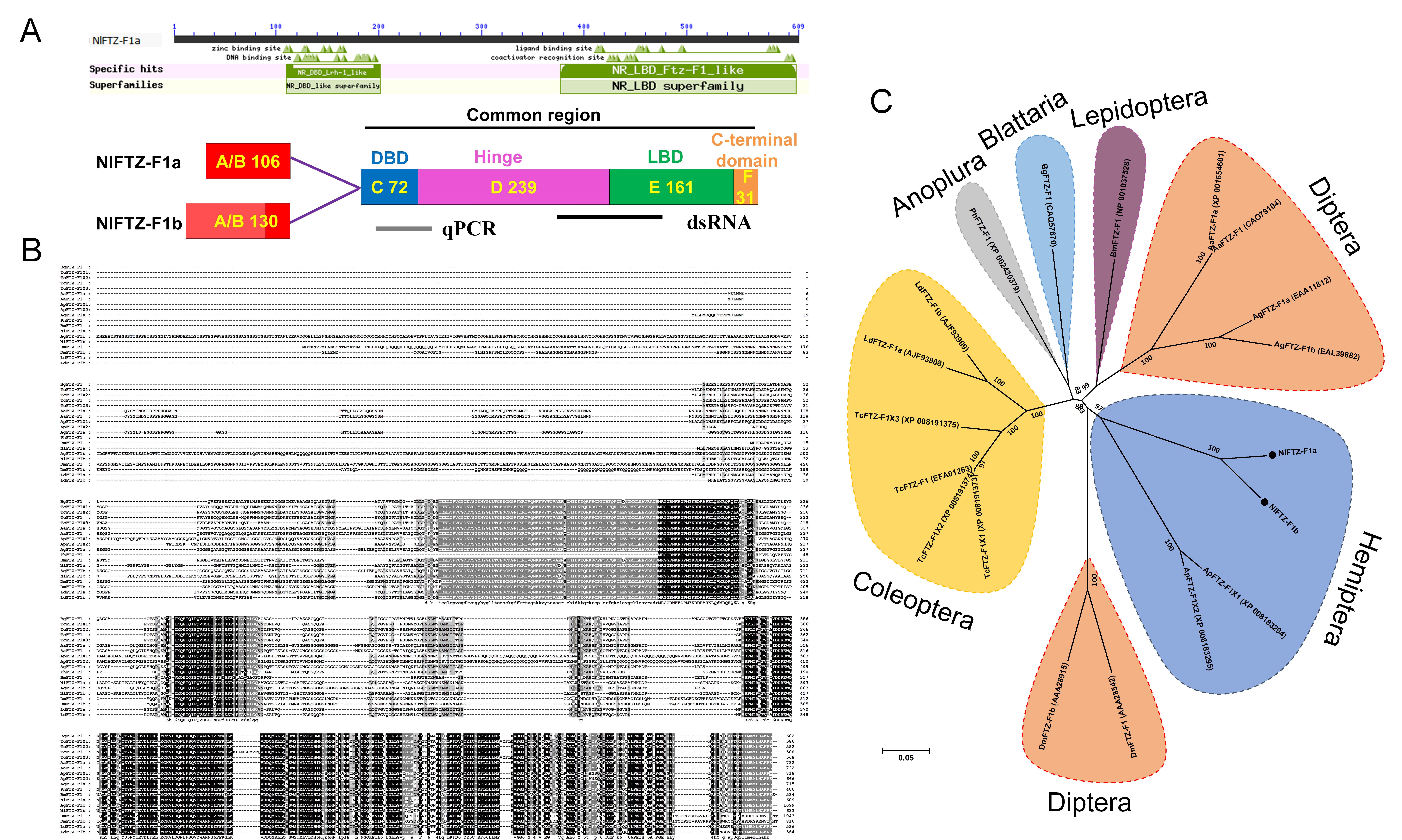


**Figure S2. Splice variants structure comparison, Sequence alignment and Phylogenetic tree of FTZ-F1.** (A) The prediction of conservative domains and comparison of different transcript structures of NlFTZ-F1. (B) FTZ-F1s from NlFTZ-F1a and NlFTZ-F1b (*Nilaparvata lugens*), PhFTZ-F1 (*Pediculus humanus corporis*, XP_002430379), TcFTZ-F1 (*Tribolium castaneum*, EFA01263), TcFTZ-F1X1 (*Tribolium castaneum*, XP_008191373), TcFTZ-F1X2 (*Tribolium castaneum*,XP_008191374), TcFTZ-F1X3 (*Tribolium castaneum*, XP_008191375), ApFTZ-F1X1 (*Acyrthosiphon pisum*, XP_008183294), ApFTZ-F1X2 (*Acyrthosiphon pisum*, XP_008183295), BgFTZ-F1 (*Blattella germanica*, CAQ57670), AaFTZ-F1a (*Aedes aegypti*, XP_001654601), AaFTZ-F1b (*Aedes aegypti*, CAO79104), AgFTZ-F1a (*Anopheles gambiae*, EAA11812), AgFTZ-F1b (*Anopheles gambiae*, EAL39882), BmFTZ-F1 (*Bombyx mori*, NP_001037528), DmFTZ-F1 (*Drosophila melanogaster*, AAA28542), DmFTZ-F1b (*Drosophila melanogaster*, AAA28915), LdFTZ-F1a (*Leptinotarsa decemlineata*, AJF93908), LdFTZ-F1b ((*Leptinotarsa decemlineata*, AJF93909). Amino acids with 100, >80, and >60% conservations are shaded in black, dark gray, and light gray, respectively. Gaps have been introduced to permit alignment. (C) Phylogenetic tree of FTZ-F1s of *N.lugens* and other insects constructed by neighbor-joining method based on amino acid sequence. The test of phylogeny was carried out using a bootstrap analysis of 1000 replications, bootstrap values > 50% are shown on each node of the tree.

**
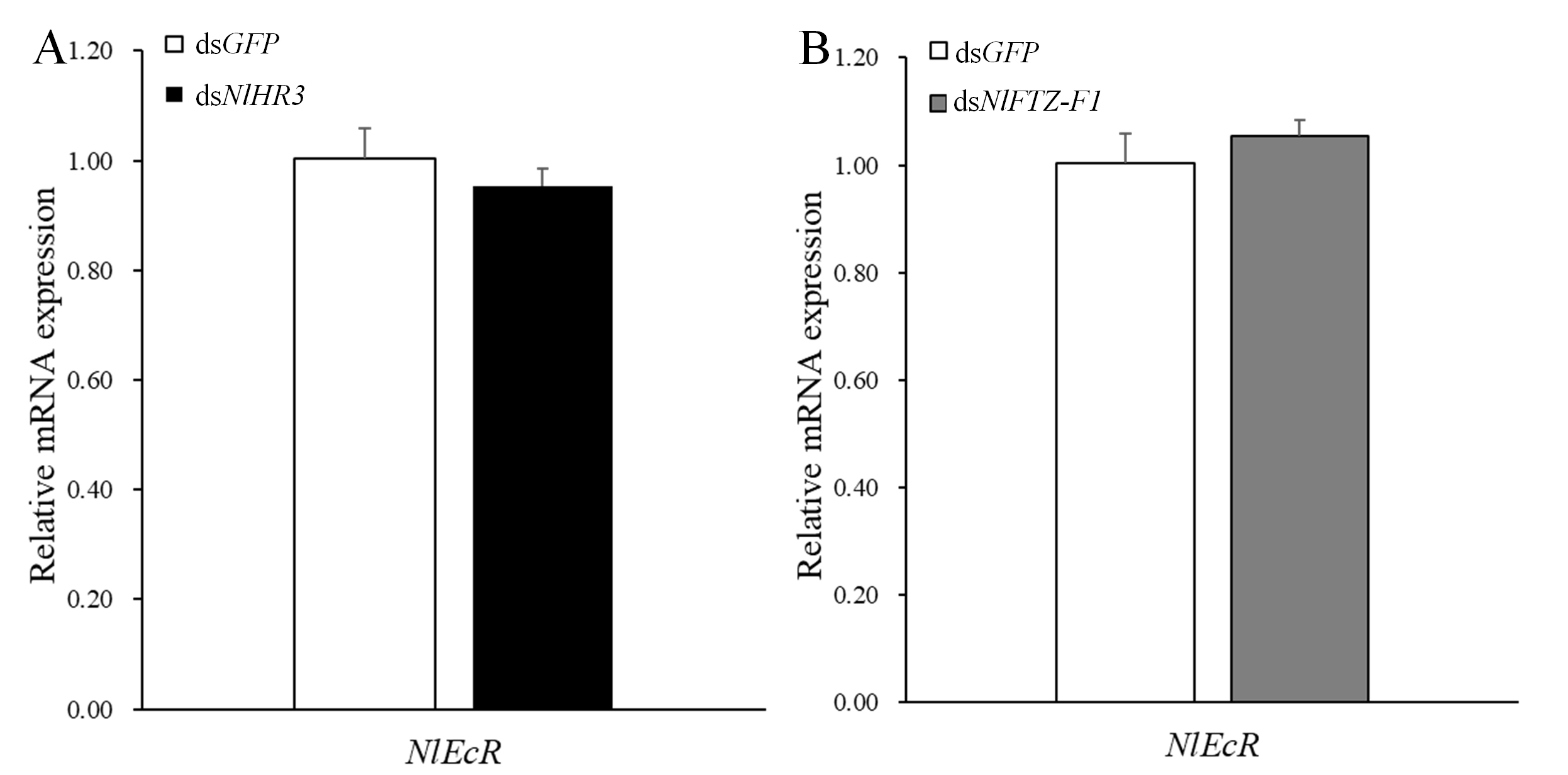
**

**Figure S3. The effect of knockdown of *NlHR3* and *NlFTZ-F1* on the expression of *NlEcR***

1. The effect of knockdown of NlHR3on the expression of NlEcR. (B) The effect of knockdown of NlFTZ-F1on the expression of NlEcR. SE was determined from three independent biological replicates, each with three technical replications.

**Table S1. Primers used for RT-PCR, dsRNA synthesis and qRT-PCR.**

| **Primer name** | **Forward sequence (5'-3')†** | **Reverse sequence (5'-3')†** | **Amplicon size (bp)** |
| --- | --- | --- | --- |
| **RT-PCR** |  |  |  |
| *NlHR3a* | CTAAGAGAGGTGCGGCTGAC | ACTATCAGCACAGGCTGCTC | 1951 |
| *NlHR3b* | AACCAGTGATATGCCAAGCA | TACCACGACACGTTGCTCAG | 1415 |
| *NlFTZ-F1a* | GTGCTGTGCTTGTTGGAGTG | GACCTTGACCTTGCTCGTGA | 2092 |
| *NlFTZ-F1b* | GCGAAGTGCAAGTGAAGTGG | GACCTTGACCTTGCTCGTGA | 2212 |
|  |  |  |  |
| **dsRNA synthesis** |  |  |  |
| ds*NlHR3* | T7-AGAGTTATGGCTGGAGTGCG | T7-TTCTCCAGTTCCAAGCGCAA | 432 |
| ds*NlFTZ-F1* | T7-AAGTACCGCCTCGAAAGGTG | T7-GTGGTGATGAGGTGGAAGGG | 474 |
| ds*NlCYP314A1* | T7-TGTTTTCGCTGGCCACATTC | T7-CACGTTTCGCCTTGTTCGTT | 460 |
| ds*NlKr-H1* | T7- CGCCAGTGAAAGTGAGACCT | T7- GAGACCGCAAGTGGTTCTGA | 498 |
| ds*GFP* | T7-CCTGAAGTTCATCTGCACCAC | T7-TGATGCCGTTCTTCTGCTTGT | 355 |
|  |  |  |  |
| **qPCR** |  |  |  |
| q*NlHR3* | AAGGAGACGTGACAGTGTGC | GGGAATTCGAGATGAGGTGT | 121 |
| q*NlFTZ-F1* | GCTACCACTATGGCCTGCTC | TTGTCGTTGGCGCATCATTT | 278 |
| q*NlCYP314A1* | TTGTTGCTCAGCCATCTTTC | TGCTGGGAATGTCGTATTGT | 102 |
| q*NlKr-H1* | AGAAAGCGCTCCAAGATGAT | GAGGCCTTGGCATAGTGAAT | 89 |

Note: T7 promoter sequences, 5**'**-TAATACGACTCACTATAGGGAGA-3**'**
